# Epidemiology, management and clinical outcomes of ICU-acquired enterococcal bacteraemias

**DOI:** 10.1186/cc14040

**Published:** 2014-12-03

**Authors:** DSY Ong, K Safdari, PMC Klein Klouwenberg, C Spitoni, JF Frencken, E Witteveen, J Horn, MJM Bonten, OL Cremer

**Affiliations:** 1 Department of Medical Microbiology, University Medical Center Utrecht, the Netherlands; 2 Department of Intensive Care Medicine, University Medical Center Utrecht, the Netherlands; 3 Julius Center for Health Sciences and Primary Care, University Medical Center Utrecht, the Netherlands; 4 Department of Mathematics, Utrecht University, the Netherlands; 5 Department of Intensive Care, Academic Medical Center, University of Amsterdam, the Netherlands

## Introduction

Enterococcal bacteraemias are associated with high mortality rates, ranging from 23 to 48% in critically ill patients. However, it remains uncertain whether these events are causes or merely markers of disease severity and mortality. Our aim was to describe the epidemiology, management and clinical outcomes of ICU-acquired enterococcal bacteraemia in comparison to other frequently isolated pathogens. Furthermore, we aimed to estimate the population attributable fraction of ICU mortality caused by enterococcal bacteraemia.

## Methods

Between January 2011 and March 2013 we included consecutive patients with an ICU length of stay of at least 3 days in two tertiary care centres in the Netherlands. ICU-acquired bacteraemia was defined as a first positive blood culture occurring at least 3 days after ICU admission. Enterococcal bacteraemias were compared to other frequently isolated pathogens with respect to patient characteristics, their management and outcomes. We used competing risk survival regression, a multistate model and cumulative incidence functions to estimate the population attributable fraction of ICU mortality due to enterococcal bacteraemia.

## Results

Out of a total of 3,108 patients, 222 (7.1%) patients were responsible for 272 events of ICU-acquired bacteraemia, of which 76 were due to enterococci, 124 to coagulase-negative staphylococci and 40 to Gram-negative bacteria. Patients with enterococcal bacteraemia were more severely ill compared to patients with bacteraemia caused by other pathogens. In comparison to patients with coagulase-negative staphylococci, those with enterococci were more frequently managed with renal replacement therapy and also had more intravascular or orthopaedic hardware. Although crude ICU mortality was higher in patients with bacteraemias due to enterococci compared to coagulase-negative staphylococci, this association disappeared after adjustment for confounders (subdistribution hazard ratio 1.04, 95% confidence interval 0.65 to 1.68). The population attributable fraction of ICU mortality due to enterococcal bacteraemia was 4.7%.

## Conclusion

Bacteraemias with enterococci occur in more severely ill patients, but their virulence seems comparable to that of coagulase-negative staphylococci. Furthermore, the population attributable fraction of ICU mortality due to enterococcal bacteraemia is low. Therefore, enterococcal bacteraemias are more likely to be markers than causes of increased disease severity and mortality.

